# Expanding perspectives and understanding relational potential: Are mutually beneficial human-animal relationships compatible with current animal agricultural practices?

**DOI:** 10.1017/awf.2024.62

**Published:** 2024-12-20

**Authors:** Erin B Ryan, Daniel M Weary, Gosia M Zobel, Jim Webster, E Tory Higgins, Becca Franks

**Affiliations:** 1 Animal Welfare Program, Faculty of Land and Food Systems, University of British Columbia, Vancouver, BC, V6T 1Z6, Canada; 2 EthicoNZ, Hamilton, New Zealand; 3 Department of Psychology, Columbia University, New York, NY, 10027, USA; 4 Department of Environmental Studies, New York University, New York, NY, 10003, USA

**Keywords:** animal welfare, farm animal welfare, human-animal relationships, intersubjectivity, positive welfare, shared reality

## Abstract

Animal agriculture employs approximately one-eighth of world’s human population and results in the slaughter of over 160 billion animals annually, representing perhaps the most extensive intertwining of human and animal lives on the planet. In principle, close, intersubjective relationships (involving shared attention and mental states) between humans and the animals in agriculture are possible, though these are infrequently studied and are unlikely to be achieved in farming, given systemic constraints (e.g. housing and management). Much scientific research on human-animal relationships within agriculture has focused upon a fairly restricted range of states (e.g. reducing aversive human-animal interactions within standard systems, toward improving productivity and reducing injuries to workers). Considering human-animal relations along a continuum, we review scholarship supporting the rationale for expanding the range of relationships under consideration in animal welfare research, given the impacts these relationships can have on both animals and stockpersons, increasing consumer demand for humane food products, and the goal of providing animals under our care with good lives. Looking toward traditions that encourage taking the perspective of, and learning from non-humans, we provide entry points to approaches that can enable animal welfare research to expand to investigate a broader range of human-animal relationship states. By showing the potential for close mutually beneficial human-animal relationships, this line of research highlights pathways for understanding and improving the welfare of animals used in agriculture.

## Introduction

Globally, each year, 160 billion terrestrial and aquatic farmed animals are killed for food (Sanders 2018; Franks *et al.* 2021), with 1.3 billion people employed in food animal systems (Thorton *et al.* 2011). Given the immense number of lives affected by the intensive human-animal relations in agriculture, understanding the potential and nature of these relationships is critically important and pertinent given the growing evidence that what is bad for animals in these environments is often also bad for humans and *vice versa* (Porcher *et al.* 2004; FAO & Brooke 2011; Porcher 2011; Pinillos 2018; Anneberg & Sandøe 2019). While mutually beneficial relationships, e.g. relationships where both parties receive and give respect, engage in reciprocity, look out for each other’s welfare, and support each other’s flourishing, are not evident or well-described in the animal welfare literature, other areas of scholarship offer novel, understudied perspectives on the human-animal relationship, providing lessons and inspiration for how the range of relationships in agriculture may be expanded and, possibly, improved. Crucially, in this tradition, we use the term ‘relationship’ in the broadest sense to refer to any intertwining or interdependency of two lives, regardless of the duration and degree of direct physical contact. Taken in the widest sense, such interdependencies can result in degrees of symmetrical or asymmetrical benefit/harm, which affects the relational quality from one of respect, mutual benefit, attention, and care, to abuse and exploitation. In this paper, we use the term ‘animal welfare’ in a straightforward sense, simply to refer to the well-being of individual animals, how well they are coping with their environment and conditions — biologically, behaviourally, and emotionally (Broom 1991; Fraser *et al.* 1997). To better understand the factors at play, we consider animals used in agriculture, as well as those in sanctuaries, private homes, zoos, and outside of direct human control. We contend that an exploration of the full range of potential human-animal relationships, including those that may be impeded in modern agricultural settings, holds promise for the field of animal welfare science to grow both its knowledge base and its capacity to improve the welfare of animals.

Our narrative review sought to describe the current state of knowledge from a variety of academic perspectives that address relationships between humans and animals, focusing mainly upon our areas of expertise, including animal welfare, psychology, animal studies, animal cognition, and animal behaviour while also touching upon themes in additional disciplines, such as rural studies, animal geography, anthrozoology, animal ethics, and animal care ethics. We began our search using key sources within animal welfare science literature and then expanded it using the papers cited and other sources recommended to us by colleagues.

This paper consists of three main sections. The first, *Farmed animal welfare*, reviews literature on what is currently known about human-animal relationships in agricultural systems, explores gaps and barriers that exist in understanding the full potential range of these relationships, and suggests that mutually beneficial relationships between humans and animals are often impeded in current systems of farming.

The second section, *Intersubjectivity*, looks beyond farmed animals, and introduces readers to approaches in scholarly disciplines outside of animal welfare science that have considered ways of engaging with animals. Intersubjectivity is defined as, “*the sharing and representing of others’ mentality*”, including mental states such as beliefs, desires, emotions, intentions, and attentional foci (Gärdenfors 2008; p 52). Scholars who study intersubjectivity emphasise joint action as a fundamental expression of shared experience and understanding between humans and animals (Konecki 2007). By shifting towards actions, the need for self-reports through symbolic language is de-emphasised and frees up the possibility of pursuing intersubjectivity with non-humans through non-linguistic means.

In the third section, we conclude with priority areas for new research on human-animal relationships and the attendant implications for animal welfare. This paper encourages reflections on current conditions and approaches to understanding farmed animals’ welfare towards the goal of transforming current farming systems in order to ensure shared realities (e.g. shared goals) between humans and animals, in diverse forms.

## Farmed animal welfare

We aim to explore the full range of relational possibilities between humans and farmed animals. This exploration seeks to enrich our understanding of human-animal relationships, generate novel data about animals and their welfare, and advance the conversation as to how to improve the lives of animals.

If imagined along a continuum, among the most negative human-animal relationships in farming are those captured in undercover videos depicting abuse or neglect of animals by farm workers. This starting place is one of extreme power imbalance and exploitation. Looking at points along the continuum away from this, relationships between humans and animals move toward mutual benefit. There are many examples of relationships that are *in the direction* of being more mutually beneficial, such as the attempts at meaningful appreciation, communication, and attention by horse whisperers like Monty Roberts. In this tradition, humans modify their behaviour to establish relationships, taking the animals’ point of view and using an understanding of their behaviour (e.g. via engagement in social contact and grooming), so that the animal may come to see the human as a trusted companion rather than a threat (Farmer-Dougan & Dougan 1999). We include this example not as the pinnacle of human-animal relationships but as promising direction. As Hineline (1986) notes, where relationships fall along the continuum of possible experiences is dependent upon the quality of interactions.

Recent research efforts in animal welfare science have made progress in understanding and addressing some of the issues related to human-animal interactions in farming, including the use of gentler handling methods (Hemsworth *et al.* 2002, 2011; Ivemeyer *et al.* 2011; Coleman & Hemsworth 2014; Tallet *et al.* 2014; Brajon *et al.* 2015). Research has also identified elements that influence how animals perceive and respond to humans, including species factors (Payne *et al.* 2016), underlying personality traits of animals, previous interactions with humans, and capacity to distinguish emotional states in humans (Waiblinger *et al.* 2006; Nawroth *et al.* 2018). Consistent with aspects of the intersubjectivity approaches (discussed below), animal welfare science has established that certain attitudes, personality traits, behaviours, and aspects of job satisfaction are associated with empathy for animals and with the animals experiencing more positive interactions for the human caregiver (Coleman *et al.* 2000; Hemsworth *et al.* 2002, 2011; Boivin *et al.* 2003; Coleman & Hemsworth 2014; Anneberg & Sandøe 2019; Rault *et al.* 2020; Acharya *et al.* 2022). Some of the same traits also correspond to increased worker well-being (Daigle & Ridge 2018). This empathy dynamic builds a feedback loop which influences the ongoing formation of attitudes of people caring for animals that impacts how they behave (Adler *et al.* 2019), and that in turn influences the responses of animals (Rault *et al.* 2020; Acharya *et al.* 2022).

Humans and at least some non-human animals are able to interpret the states of one another, strengthening the possibility that positive human-animal relationships improve the well-being of both parties (Porcher *et al.* 2004; Porcher 2011). Positive relationships are possible but may be under studied in applied animal welfare science; instead the focus has been on “*the negative end of the scale*”, including aversive states such as pain and fear (Waiblinger *et al.* 2006; p 228).

Efforts to move beyond more aversive human-animal relations and address the gaps in the research at an individual or dyadic level are limited by various impediments, likely including attitudinal (e.g. industry and farmer beliefs, and attitudes) systemic (e.g. industry and farmer goals, priorities, and management programmes), financial (Mills *et al.* 2023), physical (e.g. built environments, such as housing structures) structural and linguistic (e.g. language used), described in the following sections.

### Impediments to close relationships in agriculture

Birke *et al.* (2007) point out that worker identities (including goals and responsibilities), and the identities that workers perceive in animals (e.g. as intelligent, or not; or as dangerous or not), are constructed in part by the emotional and proximal distance between humans and animals, including how animals are managed, trained, housed, handled, and the procedures they are exposed to (Farmer-Dougan & Dougan 1999). Workers may characterise the capacities of animals in ways that can allow for the belief that an animal is not harmed by certain forms of treatment or that it is not owed greater moral regard. Such factors, especially in highly industrialised systems, can reduce the possibility of perspective-taking and limit the ability for animals to express choice, autonomy, control, or natural behaviours, which can then serve to reduce workers’ perceptions of animals’ capacities and needs.

A study by Anneberg and Sandøe (2019) illustrates how management decisions affected how workers view and treat animals on-farms. One stockperson stated, “*Where I work now, I can see they* [the sows] *do enjoy to be loose in deep bedding* [during oestrus] *so I guess it matters, but where I worked before, they were not loose during oestrus, and I never thought about it*” (p 27). Thus, structural aspects of rearing systems can impede worker ability to empathise with animals. Moreover, the inability to “*manoeuvre within the framework of conditions in which* [farm staff] *are required to work*” (Anneberg & Sandøe 2019; p 29), including a lack of appreciation from management, and a lack of control or input into how work is done, can negatively impact attitudes and behaviours towards animals (Porcher *et al.* 2004; Hassink *et al.* 2013; Anneberg & Sandøe 2019).

The built environments that animals live can also undermine the ability to forge close human-animal relationships. Housing systems can restrict behaviours, and the use of some systems (e.g. gestation crates) can make it a challenge for stockpersons to interact with and view animals as individuals (Porcher 2011; Anneberg & Sandøe 2019; Mills *et al.* 2023). Moreover, the use of management practices that are aversive to the animals (e.g. cow-calf separation, castration and dehorning without pain mitigation, routine de-beaking of chicks, use of electric prods to move animals), can encourage workers to distance themselves from the subjective experiences of the animals (Wilkie 2005; Porcher 2011; Anneberg & Sandøe 2019). Conversely, providing animals with more physical freedom and control may improve worker perception of animals’ capacities and improve job satisfaction for workers (Pinillos 2018; Johnson *et al.* 2019).

Other aspects of animal farming may impede the capacity of humans to recognise individual characteristics in animals (Wilkie 2005; Buller & Roe 2018) and the formation of human-animal attachments. For example, even if workers are motivated to attend to animals’ perspectives and individuality, demands from competing tasks with greater priority can result in worker frustration, rough handling of animals, and failure to provide materials for the expression of natural behaviours (Wilkie 2005; Anneberg & Sandøe 2019). Important to consider in these cases, is the potential for workers to experience moral injury (the negative impact on psychological wellness from events that are perceived to be ethically distressing) when standard required practices often require treatment of animals that is disrespectful (Johnson & Smajdor 2019; Williamson *et al.* 2022). The injury may be especially pronounced when the harms inflicted on animals are at the hands of workers who seek more respectful relationships. Furthermore, the division of the production chain (e.g. from farm to auction, to slaughter) sends vulnerable animals into exceedingly challenging situations where they experience variability in environments, in time spent with different workers, and in how they are handled (encountering people who likely differ with respect to their sense of responsibility toward animals) (Buller & Roe 2018). Further, work in the field of critical animal studies points to the difficulty imposed by standard housing and management practices to recognise “*the ways nonhuman animals resist what humans do to them*” (Taylor 2013; p 541). Insofar as their value is recognised, caring relationships are acknowledged mainly for their preventative role in farming systems (e.g. workers may gently handle animals to reduce incidence of injury when, for example, removing calves from cows). However, these relationships are not fully recognised as subjectively important to individual animals and objectively valuable to society (Cooke 2019, 2021; Benz-Schwarzburg & Wrage 2023). Cooke (2021) explores the ethical significance of trust in human-animal relationships within agricultural and laboratory contexts and highlights the paradox of humans intentionally building trusting connections with animals only to exploit these later. This betrayal is identified as a moral blind spot, underscoring the complexity of ethical considerations in interspecies relationships (Cooke 2021).

Together, these elements of animal production leave the potential for both shared negative and positive experiences between humans and animals significantly difficult to address. The lack of close human-animal relationships in industrial settings does not mean that these relationships cannot exist at all outside of contexts that are exploitative and where benefit is unidirectional (i.e. for humans only). What conditions might we expect to favour close human-animal relationships on farms? Aaltola (2019) summarises Weil and Murdoch’s emphasis on the important role that attention plays in shaping reality and moral action, saying, “[w]*hen paying attention to the realities of others, we also by necessity come to note their value*” (p 201). Certain kinds and practices of attention can connect us to moral action by showing us how the interests of animals may be rendered unachievable by current practices (Aaltola 2019). Further empirical work can identify which conditions best enable close relationships and how the effects of such relationships can extend to other animals and humans within the same space.

Language signals what is valued in relationships and what relationships can be expected (Wilkie 2005; Campbell 2020). Animals in agriculture are commonly referred to in relation to their status as commodities using resource language, e.g. ‘livestock’, ‘breeding stock’. These terms deindividualise animals and cause them to be seen as interchangeable, likely affecting the human-animal relationship and how they are treated. In addition, how animals respond to human handling and management is often described in mechanistic terms (e.g. improved growth rate, easy to manage; Buller & Roe 2018). Such language focuses on causal descriptions, reinforcing “*the view of animals as mere objects or vehicles of their genes and environment, pre-empting any inferences to their mental life or agency*” (Webb *et al.* 2019; p 782). In these ways, language can diminish the extent to which animals are seen to have worth beyond their instrumental value (Livingston 1994; p V). This language may also enculturate people to think and act in ways that impede engagement with animals as sentient individuals, and thus also challenge their ability to represent the interests of animals in decisions that affect their welfare (Franks *et al.* 2020).

We turn next to how we might begin to better understand and approach the perspectives of animals, looking to species and perspectives outside of farmed animals and animal welfare science.

## Intersubjectivity

Intersubjectivity in its various forms provides a mechanism for how humans can better appreciate the capacities and perspectives of others. We begin with related concepts in social psychology and then cover the methods used to understand and measure intersubjectivity in research. Within social psychology, Shared Reality (SR; Higgins 2016) is a well-researched framework for understanding how humans experience common states with others. Thus far, SR has only been explicitly studied within human-human relationships, but the theory and framework provide a perspective on the psychological mechanisms at play when humans are motivated to share interests and goals of an ‘other.’ As such, SR can provide insights into the human side of close human-animal relationships and perhaps also for non-human animals as well. With an SR lens, we explore psychological research specific to human-animal relationships, including the concept of Solidarity with Animals (SWA) (Amiot & Bastian 2017), using human-dog dyadic play as an example (Horowitz & Bekoff 2007). In the second part of this section, we turn toward perspectives on human-animal relationships that are currently outside of mainstream science, including examples of indigenous ways of understanding human relationships with animals, approaches to field research that involve taking the perspective of animals, and how language sets the boundaries of what we can expect to learn from animals and shapes our interactions with them. Relationships that encourage reciprocity and kinship, based upon listening, and guided by the interests of animals, acknowledge the power of language as a mediating factor in human-animal relationships. These relationships demonstrate the potential positive effects that connections with animals can have on our sense of identity and our understanding of animal behaviour. Importantly, insights gained from these approaches and efforts to change the nature of human-animal relationships, stand to improve the welfare of animals in numerous ways. For example, this type of work can improve our understanding of animals, enhancing our ability to detect and respond to their suffering. It also provides a more complete gauge of the harms caused to them within current systems. Additionally, it offers humans a chance to update behaviours to prioritise animal welfare in various practices. Ultimately, it encourages a shift in attention and benefits from a unidirectional flow to a more reciprocal, bidirectional one between humans and animals.

### Social psychological perspectives

SR research in human social psychology has described how close relationships develop and has identified a developmental trajectory for close relationships between humans (Higgins 2016). This body of work points to the importance of sharing experiences, intentions/goals, and joint action with others in helping build our sense of reality and identity (Rossignac-Milon *et al.* 2018). It has been suggested that similar processes are likely at play for intersubjectivity in human-animal relationships (Konecki 2007). For SR to occur, people must perceive that they are sharing with someone else a common inner state (e.g. a belief or feeling) about something (e.g. a person or an event) (Echterhoff *et al.* 2009; Higgins 2016). This process begins with ‘joint attention’. For example, “*when people meet a new employee at their workplace, they tend to create their impressions of the newcomer jointly with their colleagues, and they feel more confident in their impressions when others agree*” (Echterhoff *et al.* 2009; p 496). Thus, perceptions of reality are amplified when SR is taking place in that “*events may feel more real, sensations more clear, and interpretations more certain*” (Rossignac-Milon *et al.* 2018; p 6). Sharing reality manifests from sharing routines or practices, sharing self-guides, where both parties are able to show that they understand the rules laid out by one another, and finally, SR involves investment of the self in co-ordinated activity with those to whom one feels committed resulting in shared ways of perceiving reality (Echterhoff *et al.* 2009; Rossignac-Milon *et al.* 2018).

While Higgin’s SR model has not yet been used to investigate human-animal relationships, other research has examined human-animal relationships using similar concepts. For example, research by Horwitz and Bekoff (2007) on dyadic play between humans and dogs, investigated joint attention (a component of SR). Their work analysed non-verbal interactions between dogs and humans as manifestations of shared states. As Konecki (2007) points out, co-ordinated interactions like play bouts can be studied by asking the human involved in the human-dog dyad to describe emotional dimensions of their encounters with the animal (e.g. touching, body language, vocal expressions). Also relevant to the potential for SR between humans and non-humans is work using Qualitative Behavioural Assessments (QBA) showing that many people are able to draw inferences regarding subjective states in a range of animals (Rutherford *et al.* 2012; Jarvis *et al.* 2021).

Domestic dogs are excellent subjects to understand some of the mechanisms that allow us to share realities with animals, including the capacity to recognise states in one another. From the animals’ perspective, Turcsán *et al.* (2015) showed that dogs have the ability to recognise disgust and happiness in their owners’ facial expressions, and from the human perspective, found that children and adults were able to accurately categorise the states (angry, afraid, happy) of dogs based on acoustic cues.

The concepts of Solidarity with Animals (SWA) and empathy (i.e. acknowledgement and sensitivity to another’s experience) also overlap with the concept of SR. Amiot and Bastien’s (2015, 2017) work described the nature and the strength of human-animal interactions by examining how human perceptions of overlapping identity with other species influence the degree to which people consider animals as part of their ingroup (i.e. those with whom we share values and attitudes and who we favour with moral concern). The concept of SWA involves “*the sense of belonging, psychological attachment, and closeness toward other animals*” (Amiot & Bastian 2017; p 2) through the adoption of perspectives tuned toward what is important for the animals (e.g. their goals). Both SWA and SR focus upon outcomes of psychological closeness that humans perceive between themselves and others. Amiot and Bastien (2017) developed a measure of SWA to examine the psychology of human connections to animals and found that SWA was more predictive of positive attitudes and behaviours towards animals than other psychological scales of identification (e.g. identification with other humans or identification with nature; Stern *et al.* 1995; Leach *et al.* 2008). Amiot and Bastien (2017) point toward the implications of feeling close bonds to animals, including increasing one’s sense of obligation towards animals, consideration of their perspectives, reducing biases towards humans compared to animals, and a willingness to participate in actions to improve animals’ lives.

The idea of sharing reality with animals is perhaps most often described through concepts of emotional intelligence and empathy, which both involve the ability to relate to another’s experience, based, in part, upon the recognition “*of others as minded subjects*” (Webb *et al.* 2023). Emotional intelligence (EI) refers to “*the ability of an individual to monitor his or her emotions as well as those of others, and to use this information as a guide for thoughts and actions*” (Payne *et al.* 2016; p 115), while empathy involves the recognition of an other’s internal or subjective experiences and can thus shape the quality of the relationship (Muri *et al.* 2012). For example, Payne *et al.* (2016) suggest that dog owners scoring highest in self-reported EI are likely to have more positive relationships with their dogs (e.g. human-dog dyads presenting behaviour associated with secure attachment bonds as opposed to anxious or avoidant attachment) compared to dog owners with lower EI (Payne *et al.* 2016).

The concepts of SR, SWA and EI provide approaches that may help people working with animals develop stronger human-animal relationships. For example, these results suggest that encouraging people to identify shared goals, show empathy, and pay closer attention to non-verbal communication (e.g. co-ordinated movements) may all have benefits.

## Additional perspectives

There is considerable diversity in ways of viewing human-animal relationships; understanding various perspectives may help inform approaches to improving relationships. Of special interest are traditions in which humans are encouraged to adopt the perspective of animals; these ways of engaging with the natural world can be seen as sophisticated forms of human-animal intersubjectivity.

### A selection of indigenous knowledge-systems and approaches

Indigenous cultures are varied (Singh 2023). Here, we discuss a small sample of these approaches to human-animal relationships. Kimmerer (2013) encourages her students to approach scientific inquiry with a willingness to be taught by the subject of their inquiry; she explains that “[e]*xperiments are not about discovery but about listening and translating the knowledge of other beings*” (Kimmerer 2013; p 158), a view that contrasts scientific practices that emphasise control over research subjects and testing *a priori* hypotheses. The traditions of the Blackfoot, a First Nations tribe originating in the north west of North America, conceptualise a similar dynamic in which they regard the buffalo as their brother who teaches them how to live (Ladner 2003; Oetelaar 2014; Haggerty *et al.* 2018). Traditional Māori (indigenous Polynesian people of mainland New Zealand) ethics identifies non-human life, and also environmental features such as mountains and rivers, as people to whom one can be related (Roberts *et al.* 1995; Woodhouse *et al.* 2021). Continuity and similitude between humans and the natural world and lack of control over the environment are central to Māori understanding of the natural world (Woodhouse *et al.* 2021). As Woodhouse *et al*. (2021) report, the “*relationship between Māori and the environment is one that ties them deeply to it, further it establishes that neither animals nor the rest of the natural world, a category that includes humans, exist for the purpose of being exploited and extracted for human use*” (p 3).

The language used supports these perspectives, e.g. the use of the word ‘brother’ or ‘person’ to refer to non-humans, creates a sense of mutual dependency with attendant moral obligations rooted within a kinship paradigm. Endorsing a worldview where “*animals are not considered inferior to humans*” (Deckha 2020; p 77), indigenous scholar, Margaret Robinson describes her experience and understanding of the Mi’kmaq (a First Nations tribe originating in the north east of North America) peoples’ engagement with the natural world as being predicated on beliefs that everything is connected, alive, and in possession of an identity (including plants, water, rocks, and even geographic locations; Robinson 2014). The moral obligations of such a world view include reciprocity and respect (e.g. giving animals the necessary conditions to flourish; taking only what you can use from the natural world) and prohibit exploitation of the natural world, including animals (e.g. treating them as objects), who are perceived as brethren (Robinson 2014; Woodhouse *et al.* 2021). These shifts in language and perspective echo those of other scholars, who have argued that conceiving of other beings as ‘persons’ may help us to recognise them as ‘social subjects’ (Smuts 2006; p 125), creating the opportunity for stronger relationships.

The linguistic aspect of this ethic is important; framing in language can go in either the direction of reducing or expanding our sense of connection to the natural world, for example, language that discusses sentient animals as commodities or resources rather than kin can reduce this sense of connection. Harari (2017) explains how the introduction of agricultural, economic relations (which justify exploiting animals) to the Nayaka (present-day hunter-gatherers of south India), changed their language:

*“In the Nayaka language a living being possessing a unique personality is called mansan. When probed by the anthropologist Danny Naveh, the Nayaka explained that all elephants are mansan. ‘We live in the forest, they live in the forest. We are all mansan…So are bears, deer and tigers. All forest animals.’ What about cows? ‘Cows are different. You have to lead them everywhere.’ And chickens? ‘They are nothing. They are not mansan. And tea bushes? ‘Oh, these I cultivate so that I can sell the tea leaves and buy what I need from the store. No, they aren’t mansan.’”* (p 96).

Examples of some indigenous perspectives on human-animal relationships are valuable to look towards, showing those of us ensconced in other ontological stances toward the natural world that there is not a single, static, or settled view on human-animal relationships; that how we experience the natural world (including animals) can depend upon the quality of attention we give to it and the goals we value. This sample of certain indigenous ways of relating to animals is also included, first to acknowledge that the dominant view of these relationships in agricultural systems is neither static nor settled, and second, to illustrate how our attention might shift and our current views regarding farmed animals might deepen, towards better human-animal relationships.

### Theriomorphism and the importance of language

If humans are willing to be taught by animals, how might we go about becoming better students? Theriomorphism encourages taking the point of view of the animal you are studying (Arnet 2019); this approach may allow for insights into an animal’s experiences. In this way, humans imagine the life of an animal instead of projecting their “*mentalistic self into the life of a member or another species*” (the latter considered a drawback of anthropomorphism; Wynne 2006; p 132). Learning from another involves understanding their perspective; taking an animal’s point of view thus may facilitate learning from it (Horowitz & Bekoff 2007) and might help in the development of stronger relationships.

Adopting an other’s perspective requires communication, which between humans and animals typically takes place through non-verbal channels (Argent 2012); a challenge, perhaps, for those who believe that oral self-report is the most convincing evidence of subjectivity and a wedge for those who believe humans to be solely capable of communicating symbolic language. Indeed, even when animals have been trained to use human language, critics have expressed scepticism regarding the use of these expressions to draw inferences regarding their mental states. A key example of this dynamic is Alex, an African Grey Parrot, who learned to use over 80 words to name objects and express concepts (Pepperberg 1999; Hesse & Potter 2004). Controversially, Alex also used terms seemingly to express emotions and connection; Alex’s expression of “I love you” (spoken to Pepperberg) is seen by some to be mere imitation (Weil 2010). It is beyond the scope of this paper to determine the motivation behind Alex’s words, we encourage readers to balance two potential errors: (1) seeing the behaviours of animals as evidence of a strong human-animal relationship where none exists; and (2) ignoring strong human-animal relationships because of uncertainty as to the ‘real’ meaning of behaviours. Some uncertainty is inherent in all communication, including that among humans.

The language we use shapes our empathetic stances toward others, with both positive and negative effects. As Bastion *et al.* (2011) argued, “*while* [a]*nimals and humans share many similarities… simply thinking about these similarities does not necessarily lead to increased moral concern for animals*” (p 427). Research in many fields, including animal care ethics, critical animal studies, and anthrozoology (see, for example, the sociozoological scale; Arluke *et al.* 1996; Holmes 2021) has shown that beyond empathy, people must also value the subject of their concern and that, importantly, valuing is constructed: in part by social practices that reflect power relations, and in part by language. How people treat animals and, more broadly, how we extend moral concern toward others (including those we consider outside the realm of our social categories of inclusion) depends upon how the similarity is framed. Framing animals as being similar to humans highlights morally relevant capacities shared by humans and animals, triggering moral concern (Bastian *et al.* 2011). Conversely, framing similarities in the reverse (i.e. humans as being similar to animals) has been used as a tactic of divisive political rhetoric. Comparing marginalised human outgroups to animals who are viewed negatively, such as rats, can result in dehumanisation and abuse (Costello & Hodson 2010). Thus, attention must be given to the linguistic framing and norms embedded within social and commercial practices.

### Long-term field research

**Figure 1.**
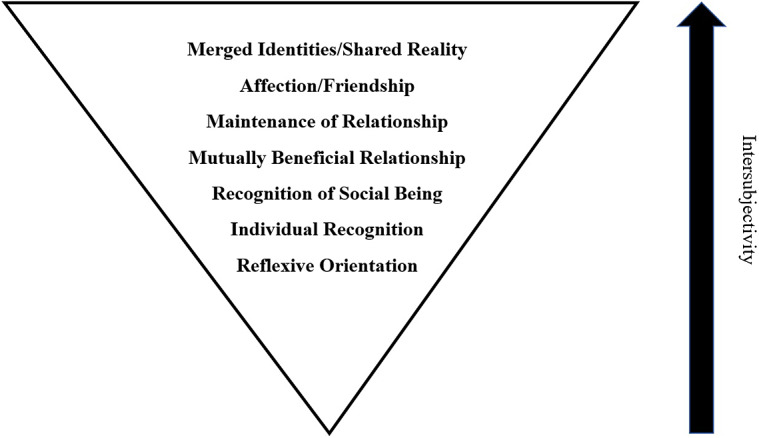
Smuts’ (2001) proposed framework for the development of close relationships between humans and other animals.

While the individual experience of the connection between humans and animals is only beginning to receive empirical attention within psychology (Amiot & Bastian 2015), for example, through the lens of attachment theory (Rehn & Keeling 2016; Hartmann *et al.* 2021), some field researchers have used previously underappreciated methodologies to forge close relationships with animals. Entering into and sustaining these close research relationships often places shared perspectives with animals at centre stage and relies upon feminist approaches that prioritise qualitative evidence and relationships, empathy for the subjective states of others, and recognition of individual expressions of personality (Fraser 2009). Though field researchers may rely upon such forms of shared reality and intersubjectivity with non-human animals to conduct their work, the central role that these relationships play in their work is sometimes underappreciated (Webb *et al.* 2023). In contrast, Barbara Smuts has written about the development and progression of her close relationships with, for example, wild baboons in East Africa (Smuts 2001). In doing so, Smuts has proposed a seven-level framework for the development of a relationships between humans and animals: (1) the animal has impersonal, reflexive, instinctual responses to the human; (2) the animal recognises the human as an individual separate from others within the human species; (3) the animal recognises that communication is possible with the human and thus recognises the human as a social being; (4) this opens the door to the development of a mutually beneficial relationship; (5) followed by maintenance of the relationship for its own sake; (6) where through joint action, affection often develops; and finally (7) awareness and identities are perceived to merge (Smuts 2001) (Figure 1).

Smuts’ approach can be seen as a complementary to Pepperberg’s, but instead of humans asking animals to learn our language, Smuts’ approach was to learn the ways of the baboons to better communicate with them. Smuts described learning to interpret subtle signals from the baboons she studied (e.g. when to halt her approach toward the baboons), and how to send readable signals (e.g. grunts, physical responses to the baboons curiosity towards Smuts) to them so that she might enter into their physical world; her work illustrates the power of verbal and non-verbal language to set up human expectations of engagement with animals we seek to have relationships with. Some scientists may view this change in behaviour as the baboons simply becoming ‘habituated’ to her presence, implying a unidirectionality in influence with the baboons adapting to her. But Smuts argued that, if anything, “*the reverse is closer to the truth*” (Smuts 2001; p 295) because she needed to habituate (alter her behaviour to match their expectations of social interactions) to gain entrance to their world. She wrote: “*The baboons remained themselves, doing what they always did in the world they had always lived in. I, on the other hand, in the process of gaining their trust, changed almost everything about myself, including the way I walked and sat, the way I held my body, the way I used my eyes and voice*” (Smuts 2001; p 295). Smuts’ willingness to see the world, as much as possible, through the eyes and ways of the baboons was rewarded with entry into their community, allowing her to experience nuances of baboon society, including social conventions (e.g. personal space, trust, and familiarity), which led her to novel considerations of human-animal relationships.

In taking the approach of habituating to the baboons (instead of asking them to habituate to her), Smuts learned “*to be more of an animal*” (p 299), with the result being that the troupe seemed to accept her into their daily lives, profoundly impacting Smuts’ sense of identity (Smuts 2001). This idea of connecting to others by acknowledging the animal within us (Serpell 2015), also appears in psychological literature on human-animal relationships, including the SWA literature discussed earlier (Amiot & Bastian 2017). The idea is that humans and animals may in some ways be able to merge identities. Smuts explains, “[a]*lthough ‘I’ was still present, much of my experience overlapped with this larger feeling entity. Increasingly the troupe felt like ‘us’ rather than ‘them*’” (Smuts 2001; p 299), including the internalised feelings of the baboons’ satisfactions and frustrations and the capacity for Smuts to predict troupe movements and where they would decide to rest at the end of the day.

The intensity of Smuts’ intentional co-ordinated activity with the baboons, in tandem with her sustained attention toward the baboons’ ways, awakened a feeling of connection to “*something larger*” (Smuts 2001; p 300). Here, again, we encounter an example where a deeper connection with animals can be enriching for humans.

For any communication system, there is the open issue of what is lost in translation, but the approach of Smuts and others can be seen as more than just information transfer; it also represents the act of reaching towards another with the intention of knowing and being known by the other. In this case, the act appeared to allow for new insights that may not have been possible using other scientific approaches (Webb *et al.* 2023). These approaches challenge more conventional methods (i.e. controlled experiments, pre-selected quantitative measures, and analysis focused on central tendencies in the data), but respond to the calls of others to consider alternative methods (Buller & Morris 2003; Porcher *et al.* 2004; Fraser 2009). Ideally, such methods attend to context, pair quantitative measures with qualitative description and narrative data, use naturalistic observation, and look to individual differences of animals (Fraser 2009). Indeed, recent calls have been made to confront the taboo of empathising with the animals in scientific studies, with some authors arguing that acknowledged and cultivated relationships between humans and animals are essential for good science and that excluding these relationships perpetuates notions of human exceptionalism and the exploitation of the animals we seek to understand (Webb *et al.* 2023). These alternative approaches involve attending to the affective states of animals and upon reflexivity on the part of the human, to understand the bi-directionality of human-animal engagement.

## Animal welfare implications

Improving relationships with animals holds the opportunity for us to better realise the potential of our humanness (Robinson 2014; Woodhouse *et al.* 2021), as the welfare of one seems connected to the other. Recent empirical evidence across several disciplines (e.g. animal welfare, economics, veterinary medicine) supports this relationship, including work that acknowledges that animals are vital supports for many people in society, including some of the most vulnerable (Deaton 2005; Walters Esteves & Stokes 2008; Serpell 2015; Siebert 2016), and that humans will sacrifice their own safety to protect the welfare of animals in their lives (Hardesty *et al.* 2013; Chadwin 2017). Further, consumers concerned with how animals in farming are treated (Buller & Morris 2003; Wilkie 2005) are increasingly willing to pay for alternative products (Bollani *et al.* 2019), including animal products from small-scale, local farms (Zomers 2020; Luymes 2021); the latter links to relationships between humans and animals (and the environment) being perceived as better on these farms (Wilkie 2005; Hopkins & Dacey 2008; Porcher 2011).

This paper has explored the complexity of human-animal relationships, and how these relationships can be consequential for those involved. Further study of human-animal relationships, should include an increased focus on structural and cultural factors that may normalise asymmetric and exploitive relationships, and thus act as a barrier in the development of mutually beneficial relationships. An emphasis on these elements could inform a shift to more equitable and compassionate directions that benefit the welfare of animals.

While there is no perfect embodiment of mutually beneficial relationships between humans and farm animals, we have presented ideas regarding approaches that might improve relationships (e.g. better attempts to listen on the part of humans, greater recognition, and responsiveness to animals’ interests). Thus, what we have proposed in this paper is for academics, farmers, industry, and others to engage in the process of aiming for something better between humans and animals, in the direction of mutual benefit. For farmers, potential interventions could include prioritising positive interactions between workers and animals as part of farm operations, incorporating perspective-taking into worker training, hiring practices that include a focus on attending to the subjective experiences of animals, and increased use of automation (e.g. robotic milking machines on dairy farms) to decrease the risk that animals and humans will come into conflict (Butler *et al.* 2012; Wildridge *et al.* 2020), and to free up time that can be reallocated to positive interactions.

Research is needed on the well-being of stockpersons and how this is affected by their relationships with the animals they are responsible for (Butler *et al.* 2012; Daigle & Ridge 2018). Future research must focus upon understanding factors related to personal background (e.g. education, addiction issues, abuse) and how these relate to capacity to engage in positive relationships (Payne *et al.* 2016). For instance, research should test the hypothesis that higher quality human-animal relationships contribute to the development of a more positive sense of identity for workers, as well as improved self-confidence. The positive impact of these relationships on the well-being of farm workers is important in its own right and may also facilitate more respectful and compassionate treatment of animals that enhances their welfare.

For animal welfare science, there is an opportunity to recast concepts of human-animal relationships with a greater emphasis on the positive needs of animals in these interactions (Rault *et al.* 2020) and to understand the potential for mutually beneficial human-animal relationships to improve animal welfare. This could involve greater efforts towards measuring welfare through the lens of relationships, and conceptualising these relationships in terms of empathy, the affective experiences of both humans and animals, and the effects of these relationships on animals, humans and the environment (following the One Welfare approach; Pinillos 2018). The study of human-animal relationships connects closely with the growing interest in One Welfare, identifying the need for science to examine the interrelated effects of relationships (Pinillos 2018).

There remains a need to understand how farming practices affect the development of relationships, including a recognition and cataloguing of expressions of resistance on the part of animals to being in certain relationships with humans. Research is required to identify where intersubjective relationships might exist on farms and how these impact animal welfare, including research on how intersubjective relationships can be identified, how they develop, and what systems or approaches encourage or inhibit their development. Studying animals and human-animal relationships in environments outside of standard systems could help researchers understand the environments that improve the welfare of animals the most. Research (Mills *et al.* 2023) asking dairy farmers to design farms from the perspectives of cows reveals the need for farmers to look toward model farms to see how management and operations could be achieved using a cow-centered approach. Research farms have the opportunity to be models that farmers can learn from and be inspired by. Research is also required on the language used to describe animals (e.g. animals’ experiences and their behaviour, and as objects or subjects), and how changes in language can lead to changes in human behaviour and animal welfare. We also need to know more about the factors that encourage people to be open to learning from animals, to take their perspective, and to see the interrelated nature of relationships (e.g. between humans, animals, and the environment). Together, these results will help inform interventions in these areas and determine ways to assess their effects on both humans and animals. In summary, the examples and literature reviewed here illustrate how a variety of perspectives, combined with imagination and engagement, can help identify ways in which animal agriculture could be fostered to transform and move toward healthier human-animal relationships.
